# Mouth-Heart Connection: A Systematic Review on the Impact of Periodontal Disease on Cardiovascular Health

**DOI:** 10.7759/cureus.46585

**Published:** 2023-10-06

**Authors:** Indu Etta, Saisravika Kambham, Khushal B Girigosavi, Binay K Panjiyar

**Affiliations:** 1 Internal Medicine, Kakatiya Medical College, Warangal, IND; 2 Internal Medicine, Dr. Bhim Rao Ambedkar Medical College and Hospital, Bangalore, IND; 3 Neurology, Dr. Vasantrao Pawar Medical College, Hospital and Research Center, Nashik, IND; 4 Internal Medicine, Harvard Medical School, Boston, USA; 5 Internal Medicine, California Institute of Behavioral Neurosciences & Psychology, Fairfield, USA

**Keywords:** hypertension, stroke, myocardial infarction, periodontitis, cardiovascular diseases (cvd), periodontal disease (pd)

## Abstract

Periodontal diseases (PDs) and cardiovascular diseases (CVDs) are highly prevalent global diseases with increasing percentages of morbidity and mortality. Both PD and CVDs independently have multifactorial causation, and emerging evidence shows an association between PD and CVDs. Periodontal diseases like gingivitis and periodontitis are chronic inflammatory conditions that eventually cause systemic inflammation, leading to many systemic diseases like rheumatoid arthritis, cardiovascular diseases, and others. In this study, we followed a systematic review approach to give an overview of the current evidence on the association between PD and CVDs. We used a relevant search strategy to retrieve articles from databases such as PubMed and Google Scholar from 2013 to July 2023. Upon applying filters and screening through titles and abstracts, we could narrow down articles to 21. On full-text screening, we selected 10 articles for in-depth analysis. This study showed a significant correlation between PD and CVDs. Poor oral hygiene, infection, and inflammation in the oral cavity lead to systemic inflammation, causing endothelial dysfunction. There are controversial views about PD acting as an independent risk factor for CVD development, as there are other risk factors such as age, gender, smoking, etc. acting as confounding factors while establishing the link between PD and CVDs. Knowledge about oral health, maintaining good oral hygiene, and proper treatment for PD could reduce the incidence of CVDs. Further research is needed to prove that PD is an independent risk factor for CVDs.

## Introduction and background

In the world of emerging diseases, cardiovascular diseases (CVDs) are the leading cause of morbidity and mortality [1]. According to the World Health Organization (WHO), around 17.9 million deaths happened in 2019 from CVDs, which accounts for about 32% of global deaths [1]. Among all the CVD deaths, 85% occurred due to myocardial infarction and cerebrovascular attacks [1]. Other cardiovascular diseases include coronary artery disease, hypertension, arrhythmias, and peripheral artery disease. Periodontal disease (PD) is a chronic inflammatory condition with multifactorial causation that destroys tooth-supporting tissues (gingiva, alveolar bone, and periodontal ligament) and eventually progresses to premature tooth loss [2]. It has been reported that periodontal disease affects about 20-50% of the population globally, and its prevalence has been increasing consistently since the last two decades [3]. People with periodontal disease may have tooth loss, excess tartar, gum inflammation and bleeding, infection, tooth mobility, decay, and gum recession with bone loss [4]. According to the literature, PD is found to have a significant association with systemic diseases like diabetes mellitus, heart diseases, systemic lupus erythematosus, and rheumatoid arthritis [5]. This study focuses on the association between PD and CVDs.

Many observational studies have reported a significant association between periodontal diseases and CVDs. According to Belinga et al., a considerable proportion of people suffering from periodontal diseases like gingivitis and periodontitis had CVDs (hypertension, heart failure, and stroke) [6]. One of the causative factors for PD is infection of the oral cavity by microbes like *Porphyromonas gingivalis*, *Aggregatibacter actinomycetemcomitans*, *Streptococcus sanguis*, *Tannerella forsythia*, *Campylobacter rectus*, *Prevotella intermedia*, *Fusobacterium nucleatum*, and *Treponema denticola* [3,6-8]. It is reported that these microbes are present in atheromatic plaques in patients with PD [6]. Two pathways establish the causation link between PD and CVD: one being transient bacteremia, while the other being increased levels of inflammatory markers in the body. Dissemination of bacteria or bacterial endotoxins like lipopolysaccharides, heat shock proteins through blood vessels leads to increased production of inflammatory cytokines, pro-thrombotic factors, and upregulation of endothelial adhesion molecules [9]. High vascularity of the oral cavity and thin, friable sulcular epithelium facilitate easy dissemination of microbes into blood [8]. Local inflammation in the oral cavity in PD manifests systemically through an increase in inflammatory markers and cytokines like C-reactive protein (CRP), tumor necrosis factor-alpha (TNF-alpha), interleukin-6 (IL-6), and interferon-gamma (IFN-gamma) [5]. These pathways cause endothelial damage and promote atherogenesis that progresses to CVDs.

Periodontal disease and cardiovascular diseases share common risk factors such as smoking, diabetes mellitus, age, socio-economic status, obesity, stress, poor nutrition, family history, and immunosuppression [8]. These risk factors act as confounders while studying the association between PD and CVDs. Evidence supports that CVDs like infective endocarditis occur due to bacteremia after dental procedures, and physicians give antibiotic prophylaxis after every oral procedure [10,11]. The oral cavity is a harbor for millions of microbes; everyday brushing and chewing allow them to translocate into blood. Thus, oral hygiene is of utmost importance to prevent such catastrophes.

This study evaluates the available evidence on the association between periodontal disease and CVD by adopting a systematic review approach.

## Review

Methods

**Figure 1:**
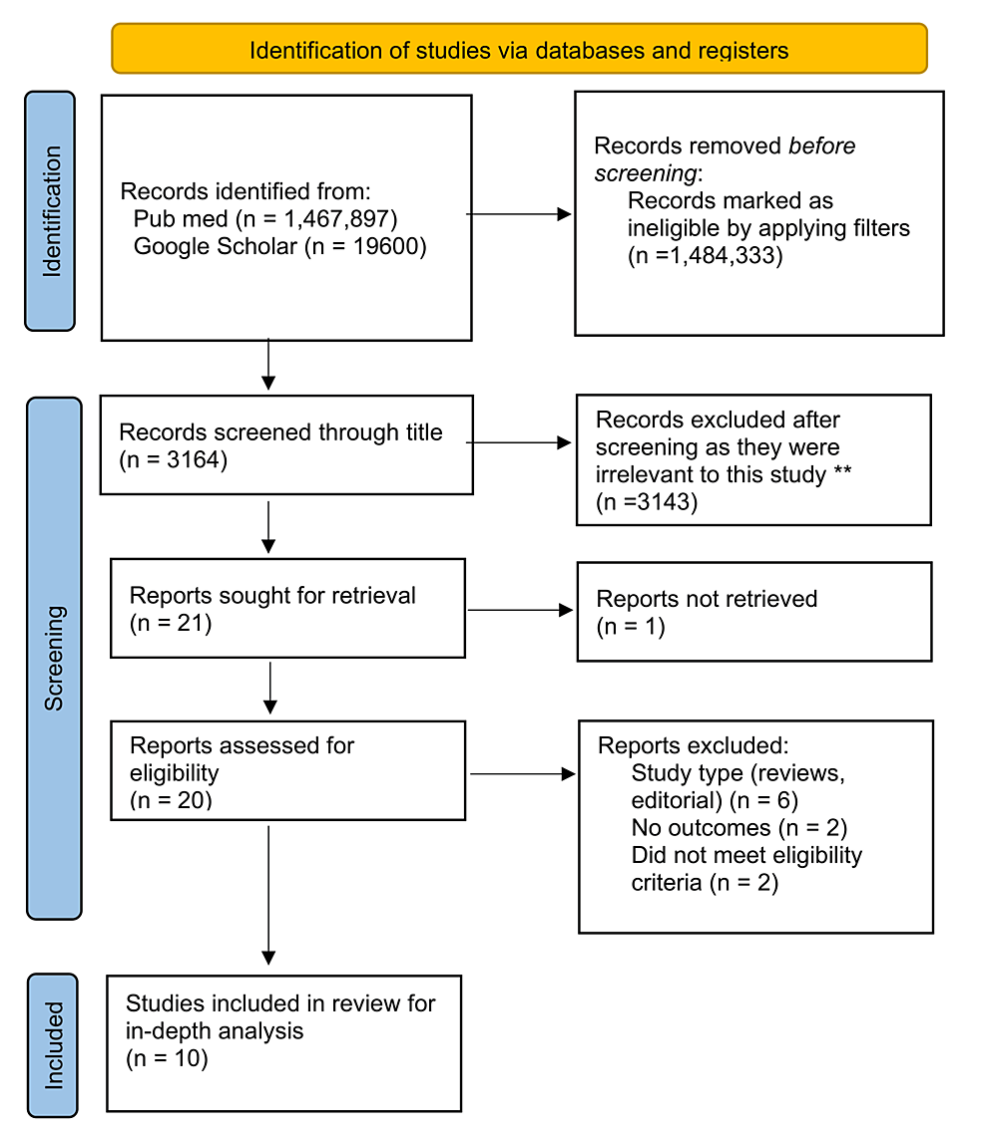
PRISMA flowchart showing the study selection process for the systematic review. PRISMA: Preferred Reporting Items for Systematic Reviews and Meta-Analyses.

This review focuses on studies evaluating the association between periodontal diseases and cardiovascular health. The review follows the guidelines for Preferred Reporting Items for Systematic Reviews and Meta-Analyses (PRISMA) [12] for 2020. Figure 1 shows the PRISMA flowchart, which depicts the process of article selection and exclusion. Data collection for this review is taken from published papers, eliminating the need for ethics approval.

Search Strategy

We used the population, intervention/condition, control/comparison, and outcome (PICO) strategy to frame a research question. Using keywords and MeSH terms, we formed a search strategy and used it to conduct searches on databases like PubMed (including Medline) and Google Scholar. The detailed search strategy used and the results obtained are mentioned in Table 1.

**Table 1:** Showing search strategy, search engine used and number of articles found.

S. no.	Database	Search strategy	Search results
1.	PubMed	((((periodontal disease(Title/Abstract)) OR (cardiovascular health(Title/Abstract))) OR (periodontal disease(MeSH Terms))) OR (cardiovascular(MeSH Terms)))	1,467,897
2.	Google Scholar	Periodontal disease AND Cardiovascular health OR Heart OR Cardiovascular	19,600

Systematic Literature Search and Selection Criteria

We used filters like the publication year, full-text articles, human studies, age above 19 years, and English texted articles as primary filters in PubMed. Duplicates were removed, and selection criteria were applied for the remaining articles. We excluded articles that did not meet the selection criteria. Studies such as randomized clinical trials, case-control studies, cohort studies, systematic reviews, and meta-analyses were included, while studies such as review articles, letter to editorial, and animal models were excluded. The inclusion and exclusion criteria used in the review are depicted in Table 2. We then independently screened through the abstracts and full text for the remaining articles to find their relevance to the present study.

**Table 2:** Showing the inclusion and exclusion criteria adopted for the study.

Inclusion criteria	Exclusion criteria
Human studies	Animal studies
Articles published between 2013 and 2023	Articles published before 2013
Adults above 19 years	Population below 19 years
Both male and female studies	Gender-specific studies
English texted studies	Non-English texted studies
Free papers	Articles that need to be purchased

Quality Appraisal

We employed certain quality assessment tools to assess the quality of papers chosen after screening and evaluate their reliability for this review. Table 3 shows the tools for quality assessment of different types of studies.

**Table 3:** Showing tools used for quality appraisal. PRISMA: Preferred Reporting Items for Systematic Reviews and Meta-analyses; SANRA: Scale for the Assessment of Non-Systematic Review Articles.

Type of study	Tools used
Randomized clinical trial	Cochrane bias tool assessment
Non-randomized clinical trials and observational studies	Newcastle-Ottawa tool
Systematic reviews	PRISMA checklist
Other studies	SANRA checklist

Results

We got 14,87,497 articles using the search strategy in PubMed and Google Scholar. We excluded 14,84,333 after applying primary filters like text availability, human-specific studies, associated data, age limit, and English texted studies on PubMed and Google Scholar. From the remaining 3164, we excluded 3143 studies after finding duplicates, screening through titles/abstracts and applying eligibility criteria. We thoroughly evaluated the remaining 21 studies and found 10 studies appropriate for our study. At last, we performed a quality check on 10 articles that met all the eligibility criteria. Table 4 shows all the articles that we included in our systematic review.

**Table 4:** Summary of the results of the selected papers. MI: myocardial infarction; PAD: peripheral artery disease; CHD: coronary heart disease; AF: atrial fibrillation; CVD: cardiovascular disease; PD: periodontal disease; DPSI: Dutch periodontal screening index; OR: odds ratio; RR: risk ratio; CAL: clinical attachment loss; BMI: body mass index; BOP: bleeding upon probing; PI: Plaque index; CI: confidence interval; CEJ: cementoenamel junction; NA: not available; CVA: cerebrovascular accident.

Author/year	Country	Study design	PD diagnosis	Outcome	Risk factors	Conclusion
Larvin et al. (2021)	UK	Meta-analysis	Clinical and self-reported	Stroke, MI, PAD, CHD, AF, hypertension, and any CVD	Smoking (not adjusted)	Increased risk of CVD in patients with PD, with risk more pronounced in males and patients having severe PD.
Belinga et al. (2018)	Cameroon	Prospective observational study	DPSI scoring	Hypertension, heart failure, and stroke	Age, gender, diabetes mellitus, and smoking (not adjusted)	A significant association between gingivitis (OR = 4.30) and periodontitis (OR = 2.87) with CVD. This study suggested periodontal treatment needs in 431 patients (77.2%).
Yang et al. (2018)	China	Meta-analysis	Clinical diagnosis or self-reported through a questionnaire.	PAD	Age, sex, diabetes, hypertension, dyslipidemia, smoking, and education (adjusted)	This study found a significant risk of PD in PAD patients than non-PAD patients (RR = 1.70). Patients with PAD had more missing teeth than controls, and there is no statistical difference in CAL in cases and controls.
Xu et al. (2017)	China	Meta-analysis	Self-reported diagnosis or clinical/radiological/microbiological assessment.	MI	Age, gender, smoking, exercise, alcohol, BMI, hypertension, diabetes, cholesterol, education, and family history of MI (adjusted)	This study showed a statistically significant association between PD and MI (OR = 2.02).
Reichert et al. (2016)	Germany	Longitudinal cohort study	Clinical dental assessment (BOP, PI, PD, and CAL).	Any CVD	Age, diabetes, and history of CVDs (adjusted)	The association between severe periodontitis and CVD could not be proven in univariant and multivariant analyses. There was no statistical significance after adjusting risk factors.
Humphrey et al. (2008)	USA	Meta-analysis	Periodontal indexes were calculated from periodontitis, gingivitis, missing teeth, and bone loss.	Any CVD	Age, sex, hypertension, diabetes, smoking, BMI, education, family history of CVD, vitamin E, and alcohol	This study evaluated the statistical significance of the association between tooth loss (RR = 1.34, 95% CI 1.10-1.63), gingivitis (RR = 1.35, 95% CI 0.79–2.30), and CVD.
Samani et al. (2013)	Iran	Case-control study	Periodontal index score (assessing gingival inflammation and depth of gingival sulcus associated with CEJ).	MI	NA	This study showed that people with a loss of more than 10 teeth are at high risk of developing MI (OR = 2.727).
Fagundes et al. (2019)	Brazil	Meta-analysis	CAL, probing depth, self-reported diagnosis.	Stroke	NA	This study performed subgroup analysis showing increased risk of CVA in patients with periodontitis in case-control (OR = 2.31, (1.39, 3.84)) and cohort studies (OR = 1.88, (1.55, 2.28)).
Muñoz Aguilera et al. (2020)	UK	Meta-analysis	Confident and non-confident definition of periodontitis	Hypertension	NA	Increased severity of PD is positively correlated with hypertension.
Zhan et al. (2023)	China	Cross-sectional study	Periodontal parameters (BOP)	Hypertension	Sex, age, smoking, income, education	41% of cases with hypertension and 28% of normotensive cases had severe periodontitis (stages III and IV).

A total of 10 studies were recruited after thorough screening. The included studies were published between 2013 and 2023. Three of the selected studies are China-based, two from the UK, and the rest from Cameroon, Germany, Brazil, the USA, and Iran. Of all the 10 studies included, six are meta-analysis studies, while the remaining four are prospective observational studies, longitudinal cohort studies, case-control studies, and cross-sectional studies. All the studies included both men and women; gender-specific studies were excluded as part of the eligibility criteria. In most of the studies, risk factors were adjusted. Among the studies included, four of them evaluated the association between PD and any CVD, while others assessed the association between PD and specific CVDs such as PAD, MI, hypertension, and stroke.

Discussion 

In this systematic review, we found a significant association between PD and CVD, which proves the impact of poor oral hygiene on cardiovascular health. According to Larvin et al., the PD population had a significantly higher risk of CVDs than the non-PD population (RR = 1.20, 95% CI: 1.14-1.28) [3]. In a Cameroon-based prospective observational study, there were statistically significant results, with about 28.9% of patients suffering from PD having CVDs [6]. Having said that, some findings are contradictory to the earlier-mentioned findings. In a longitudinal cohort study by Reichert et al., the outcomes during the follow-up of patients with PD were quantified, and their association with PD was not statistically significant in both univariate and multivariate analyses. They concluded that periodontitis was not an independent risk factor for adverse events in patients with CVDs [13].

The impact of oral health on cardiovascular well-being can be explained by many pathways to establish the causal link between CVD and PD. Gingiva is usually composed of many microbes, such as *Streptococcus sanguis*, *Streptococcus mutans*, *Streptococcus oralis*, *Actinomyces naeslindii*, *Actinomyces odontolyticus*, *Vellionella parvula*, and *F. nucleatum* in supra and subgingival spaces [14]. Poor oral hygiene, plaque formation, or any other condition triggers gingival inflammation, which changes the microbial composition to gram-negative bacilli and anaerobes such as *P. gingivalis*, *T. forsythia*, *T. denticola*, *Selenomonas noxia*, *A. actinomycetemcomitans*, *C. rectus*, *spirochetes*, and *P. intermedia* [14]. Bacterial colonization further aggravates the inflammation, which leads to the deepening of the gingival sulcus and the formation of a periodontal pocket, as well as the loss of supporting tissues and alveolar bone [14]. Eventually, periodontal inflammation causes systemic inflammation, which could be induced by bacteria or inflammatory mediators [15]. Translocation of bacteria, bacterial endotoxins, and increased inflammatory markers like IL-1, IL-6, IFN-gamma, TNF-alpha, CRP, haptoglobin, and fibrinogen initiate the catastrophic pathway.

According to Samani et al., there is an increased risk of MI in patients who lost more than 10 teeth (OR = 2.73). They found a significant difference in the mean Periodontal Disease Index (PDI) among the case and control groups, with more risk of MI in cases with PDI>4 (OR = 7.87) [14]. In a meta-analysis by Xu et al., there was a statistical correlation between PD and MI, with patients having PD at a one-fold increased risk of MI [14]. According to Larvin et al., the association between MI and PD was not statistically significant, though there was an increased risk of MI [3]. In a cohort study by Reichert et al., spillage of microbes and bacterial endotoxins like lipopolysaccharides trigger the release of inflammatory mediators and pro-thrombotic factors, which increases the chance of atherosclerotic plaques and progression of which leads to MI [16,17].

According to Fagundes et al., there is an increased chance of stroke in patients with periodontitis (RR = 2.31 (95% CI = 1.39, 3.84)) [18]. On subgroup analysis based on the type of study, cohort studies (RR = 1.88 (1.55, 2.29)) and case-control studies (RR = 2.72 (2.00, 3.71)) showed an increased risk of stroke and ischemic stroke, respectively [18]. In a meta-analysis where any CVD is the common endpoint for evaluation of the impact of PD on cardiovascular health, the risk of stroke (RR = 1.24; 95% CI:1.12-1.38) was highest among other CVDs [3]. In a Cameroon-based prospective observational study, stroke was found in only 5% of all the CVDs identified among PD patients [6]. This difference in the incidence of stroke among PD cases can suggest that PD is not an independent risk factor for stroke, though the results are statistically significant. Inflammation increases the risk of stroke with increased inflammatory markers like CRP, IL-6, and lipoprotein association phospholipase A2 [19]. Evidence shows that PD is associated with increased intima thickness of the carotid artery and the formation of carotid calcifications [8].

A systematic review and meta-analysis by Muñoz Aguilera et al. reported a positive association between PD and hypertension (an increase in the severity of PD is associated with increased mean systolic and diastolic blood pressures) [20]. In a cross-sectional study by Zhan et al., severe periodontitis (stages III and IV) was associated with hypertension, and there was no significant difference in periodontal status in hypertensive and normotensive patients with increasing age [21]. In a Cameroon-based prospective observational study, 87.6% of identified CVDs in PD patients were hypertensive [6]. Local inflammation in the oral cavity leads to systemic inflammation, causing endothelial dysfunction that affects the regulation of hypertension [22]. Increased reactive oxygen species in periodontitis are associated with vascular inflammation and vasoconstriction [22].

In a systematic review and meta-analysis on the impact of periodontitis on PAD, there is a significant difference in missing teeth between PAD and non-PAD patients, proving the statistical association between periodontitis and PAD [5]. Thus, through this review, we could derive the association between PD and CVDs such as MI, stroke, hypertension, and PAD.

There are controversial views regarding PD as an independent risk factor for CVDs. All the studies conducted in this arena mentioned other risk factors like age, sex, smoking, obesity, socio-economic status, and alcohol as confounding factors while evaluating the link between PD and CVDs. Hypertension risk was increasing in severity of periodontitis, and this association was more among individuals aging between 35 and 44 years [21]. According to Larvin et al., men with PD had a high risk for CVDs (RR: 1.16, 95% CI: 1.08-1.25) [3]. In a cross-sectional study by Zhan et al., among all the current smokers, the prevalence of PD is higher in hypertensive (76.2%) than in normotensive (68.1%) individuals [21].

Oral health could be easily affected by many factors, such as an improper diet, alcohol intake, smoking, and poor oral hygiene [23]. Poor oral health overtime influences the quality of life by affecting the cardiovascular system. Good knowledge of oral hygiene and its impact on health is essential in order to be determined about maintaining proper oral hygiene. According to Farsi et al., females and medical faculties have better knowledge of oral health than males and non-medical faculties [24]. Lower educational qualifications and low socio-economic status also have an effect on oral health knowledge [25]. A larger proportion of people do not know the impact of oral diseases on systemic health [24]. Thus, spreading knowledge about practicing good oral health is highly important in order to prevent oral diseases and maintain a good quality of life.

In a randomized controlled trial by Paju et al., clarithromycin showed beneficial effects in the prevention of recurrent cardiovascular events in non-PD patients, which suggests that oral cavity infection could lead to cardiovascular morbidity [26]. In a cohort study by Holmlund et al., patients suffering from periodontitis were given treatment, and the treatment effect was categorized as good or poor responders. Poor responders had a higher risk of getting CVDs than good responders [27]. Thus, timely treatment for periodontal disease could reduce the risk of CVDs.

There has been a lot of heterogeneity in the studies because of study type (cross-sectional study, cohort study, and case-control study), study region, confounding factors (gender, smoking, and others), and PD diagnosis method (CAL, missing teeth). All these factors are reported to affect the risk of PD on CVDs. According to Yang et al., there was a statistically significant association between missing teeth and PAD, while CAL did not show any association with PAD [5]. In a study, subgroup analysis based on study type showed that the high risk of MI in PD patients is statistically significant in cross-sectional and case-control studies while marginally significant in cohort studies [16]. Heterogeneity in results due to these factors is a limitation in evaluating the impact of PD on CVDs. Thus, there is a need for further research to prove that PD is an independent risk factor for CVDs by limiting confounders and other factors.

## Conclusions

This systematic review identifies a positive correlation between PD and CVDs. Transient bacteremia and local inflammation are the causal links for this association. Poor oral hygiene, plaque formation, and infections initiate the local inflammation in the oral cavity that eventually spreads to the blood. The independent association of PD with CVD is yet to be established. There are many confounders like gender, smoking, and others that need to be taken care of while performing studies in this arena. We could find a significant association between periodontal disease and CVDs like MI, PAD, hypertension, and stroke. Proper oral hygiene and adequate treatment for periodontal disease could lower the risk of CVDs. There is a need for further research to know whether or not PD is an independent risk factor for CVDs.
